# Women as Organisers

**Published:** 1900-09-01

**Authors:** 


### The Hospital

A Journal of

The Medical Sciences and Hospital Administration.

Vol. XXVIII.—No. 727.] Edited by Sir Henry Burdett, K.C.B., and Solomon C. Smith, M.D., M.R.C.P. [September 1, 1900.

### Women as Organisers.

The Countess of Warwick, who of late years has become known in connection with various schemes for the better accomplishment of many forms of women's work, has contributed to the last number of the *Observer* an interesting article on organisation, considered especially from a feminine point of view, and in relation to feminine capacities. Lady Warwick appears inclined to maintain that men, on the whole, have made a terrible mess of their attempts in this direction; and that the time has come for them to stand aside, and to give a fair field to women, who, in her own words, have been kept too long at fielding, and who now desire to take their places at the wickets. The Countess does not reveal her plans, nor say exactly what she and her fellow-workers propose to do, but tells the world in general to wait and see. Her case seems largely to depend upon the assumption that England, relatively to some Continental countries, does not now hold her former position of uncontested and uncontestable industrial superiority; and she tells us, truly enough, that many things are made in Germany for our consumption which might be made at home. The fact would doubtless be important if our own people were suffering from lack of employment; but, as long as this is not the case, it is probably sound economy that the requirements of the world should be supplied by different nations in accordance with the respective facilities of their peoples for doing this or that; and that the German aptitudes for the cheap and nasty may be cultivated to any reasonable extent without affording us occasion for grave alarm.

Many of our great business establishments, such, to take a single example, as the combined Armstrong and Whitworth Companies, afford ample reason for believing that the organising power of our male population is in no way diminished, and that our captains of industry are as masterful and as enterprising as ever. We cannot admit that Lady Warwick has any real ground for her pessimism, and we do not like her simile derived from cricket. We would rather go to some other description of game, even although it were still less feminine in character, in which both sides could be in the field at once. With this proviso, we are in hearty sympathy with her ambitions, and are quite prepared to admit that her clients are likely to become the equals, although not the rivals, of men.

The whole question is one in which the medical profession is deeply interested, because it can only be solved in accordance with the facts of physiology, as these are rendered more and more clear to us. Lady Warwick is impatient, perhaps justly impatient, of the distinctions which have often been drawn between the male and the female capacities for work of various kinds. There can be little doubt that differences which have been regarded as fundamental have been the mere consequences of opportunities and surroundings. The great Lipsius bequeathed his doctor's gown to an image of the Virgin Mary, and it was said that, for the first time in his life, he had been guilty of bad Latinity. He had put together the feminine gender and the masculine *case*. Very much the same thing has been done by theorists who have endeavoured to make women the rivals of men in their own lines, and have not perceived that the differences between the sexes, whether they be inherent and eternal, or merely such as may be worn away by time and altered circumstances, are yet sufficiently marked and real, at any particular period of history, to imply the greater fitness of each for certain pursuits and occupations. Lady Warwick appears to think that the secret of organisation is originality, by which, in relation to her subject, she would presumably mean the power of doing old things in a new way. We should be more inclined to look for it in the power of correctly adjusting means to ends, so as to avoid waste on the one hand and inefficiency on the other. On this assumption the success of an organiser is likely to be in proportion to his or her comprehension, first of the precise ends to be attained, next of the most economical methods of attainment. If this be so, mastery of the subject-matter becomes a primary requisite, and there are undoubtedly subjects which, in the present stage of civilisation, are more easily mastered by women than by men. The sagacity required for selecting these subjects, and for dealing with them when selected, are the qualities which Lady Warwick should seek to encourage and to develop. We will venture, albeit mere men, to suggest one in which there is practically unlimited scope for the exercise of female talent and feminine influence, and by which, if it were but adequately dealt with, the comfort of mankind might be immeasurably promoted. Could not Lady Warwick initiate some movement which would conduce to the better organisation of the domestic service that is required in countless middle-class households? an employment over which at present

"Chaos umpire sits,
And by decision more embroils the fray."

